# Fetal Macrosomia Among Non-diabetic Women: Our Experience in a Developing Country

**DOI:** 10.7759/cureus.26763

**Published:** 2022-07-11

**Authors:** Tanveer Shafqat, Laila Zeb, Sumaira Yasmin

**Affiliations:** 1 Obstetrics and Gynaecology, Lady Reading Hospital Medical Teaching Institute, Peshawar, PAK

**Keywords:** gestational age, parity, bmi, fetal macrosomia, prevalence, caesarean section

## Abstract

Background

The prevalence of fetal macrosomia varies worldwide. Its trend has increased over the past decades in many developed nations. It is associated with various maternal and fetal complications. The information regarding the frequency of fetal macrosomia among non-diabetic women is limited in resource-limited countries such as Pakistan. Therefore, this study aimed to determine the number of fetal macrosomia cases among non-diabetic women.

Methodology

This was a cross-sectional study conducted in a tertiary care hospital in Peshawar, Pakistan. A total of 119 pregnant women were enrolled in the study. All pregnant women aged 15 to 45 years who had singleton pregnancies with any parity or gravida and a gestational age of ≥37 weeks were included in the study. Pregnant women with underlying chronic systemic disorders such as diabetes mellitus, gestational diabetes mellitus, hypertension, renal or cardiac disorders, and sickle cell anemia were excluded from the study. Women who did not consent to participate and those with a gestational age of ≥42 weeks at the time of delivery were also excluded from our study. Based on a 5.2% prevalence of fetal macrosomia in the general population, the sample size was calculated using the World Health Organization calculator at a confidence interval of 95%, absolute precision of 0.05 with anticipated population proportion, and a 4% margin of error. The required sample size was calculated at 119. The chi-square test was applied. P-values of ≤0.05 were considered significant.

Results

Out of 119 participants, fetal macrosomia among non-diabetic women was seen in 10 (8.4%) cases. The mean age of patients in our study was 29.80 ± 4.33 years. The mean gestational age was 36.05 ± 1.31 weeks, whereas the mean body mass index of participants was 29.17 ± 2.36 kg/m^2^. Post-stratification, spontaneous vaginal delivery was the only significant variable with a P-value of <0.05 in our study.

Conclusions

The number of fetal macrosomia among non-diabetic women in our study was 10 (8.4%). Because this was a single-center, hospital-based, cross-sectional study, we need to conduct large multi-centered randomized controlled studies to identify the actual prevalence of fetal macrosomia in non-diabetic women in our population.

## Introduction

Fetal macrosomia is associated with numerous neonatal and maternal complications such as birth asphyxia, clavicle fractures, and brachial plexus injuries in the fetus. Maternal complications include shoulder dystocia, postpartum hemorrhage, infection, large perineal tears, cesarean delivery, and thromboembolic events [1,2]. Multiparity, a history of diabetes mellitus (DM) and previous fetal macrosomia, gestational diabetes mellitus (GDM), obesity, gestational age of 40 weeks, and maternal age of 30-39 years have been associated with fetal macrosomia [3]. This is a challenging problem even for an experienced obstetrician as it adversely affects maternal and neonatal health [4].

Fetal macrosomia, defined as a fetus weighing more than 4,000 to 4,500 g (according to National Institute for Health and Care Excellence guidelines and American College of Obstetricians and Gynecologists guidelines), is a global problem, and its prevalence has increased over the past decades in many developed nations [5]. A systematic review performed in 2019 regarding neonatal and maternal complications associated with fetal macrosomia found that it was associated with serious neonatal and maternal issues [6]. A case-control study was conducted by Melamed et al. to determine the impact of a false diagnosis of macrosomia (ultrasound estimation of fetal weight) on maternal and neonatal outcomes. The study concluded that a false diagnosis of macrosomia increases the risk of cesarean section with a high possibility of maternal and neonatal complications. The prevalence of fetal macrosomia varies globally. Ethnicity, race, lifestyle, and underlying systemic disorders such as DM play an important role in the occurrence of fetal macrosomia. The most recent data have confirmed that the prevalence of fetal macrosomia in the United States is 8% of all live births [7]. In 2011, a study conducted in a tertiary care hospital in Saudi Arabia revealed that the prevalence of fetal macrosomia is 4.5% [8]. A study performed in China confirmed that an increase in the prevalence of fetal macrosomia is due to an increase in net gestational weight gain [9]. Earlier, a study aimed to establish the prevalence of fetal macrosomia among non-diabetic women found that the prevalence among non-diabetic women was 8.9%. The prevalence of fetal macrosomia in Belgium was found to be 8.63% [10,11].

In this study, we hypothesized that the number of fetal macrosomia cases among non-diabetic women is increasing in Pakistan. Information regarding fetal macrosomia among non-diabetic women and its associated complications is limited in developing countries like Pakistan due to the lack of adequate data collection and follow-up. Therefore, this study aimed to determine the number of fetal macrosomia cases among non-diabetic women.

## Materials and methods

This was a single-center, cross-sectional study conducted in a tertiary care hospital (Lady Reading Hospital Medical Teaching Institute, Peshawar, Pakistan) from September 8, 2020, to March 7, 2021. A total of 119 patients were enrolled in the study. The study was approved by the Institutional Research and Ethical Board of Lady Reading Hospital Medical Teaching Institute (protocol number: 274/2020). Written informed consent was obtained from participants included in this study.

Inclusion criteria

All pregnant women aged 15 to 45 years who had singleton pregnancies with any parity and a gestational age of ≥37 weeks were included in the study. The number of times a woman gets pregnant is termed gravida; it can be primigravida (first-time pregnancy) or multigravida (more than one time). The number of times a woman has given birth to a fetus (alive or stillbirth) with a gestational age of 24 weeks or more is termed parity; it can be primiparous (first time) or multiparous (more than one time). The mode of delivery was categorized into spontaneous vaginal delivery (SVD) or cesarean section. Mothers who gave birth without requiring any surgical intervention were termed SVD, whereas those who gave birth by a surgical intervention were termed cesarean section.

Exclusion criteria

We excluded pregnant women with pre-existing DM or GDM diagnosed by the oral glucose tolerance test (OGTT). We also excluded women with associated chronic medical conditions such as hypertension (HTN), renal or cardiac disorders, and sickle cell disease. Women with multiple pregnancies diagnosed by ultrasound and a period of gestation of ≥42 weeks at the time of delivery were excluded. Further, those who did not consent to participate in the study were excluded.

Factors examined included maternal age, gestational age of participants, gravida, parity, body mass index (BMI), previous history of macrosomia, and mode of delivery. We considered fetal macrosomia in all newborns who had a birth weight of ≥4,000 to 4,500 g. Detailed history, clinical examination, and laboratory investigation were performed to exclude DM. The non-diabetic status of participants was confirmed from the antenatal records. Pregnant women with fasting plasma glucose (FPG) of 5.3 mmol/L, two-hour plasma glucose of 6.4 mmol/L after a meal, or HbA1C of <6.5% were considered non-diabetic. Fasting blood sugar and two-hour post-meal was repeated during their stay in the hospital. Weight and height were assessed in all patients and were graded according to the World Health Organization (WHO) protocol. BMI of pregnant patients was categorized into those with a BMI of 25-30 kg/m^2^ (overweight) or >30 kg/m^2^ (obese).

The questionnaire was developed based on a previously published paper. Based on a 5.2% prevalence of fetal macrosomia in the general population, the sample size was calculated using the WHO calculator with a confidence interval of 95%, absolute precision of 0.05 with anticipated population proportion, and a 4% margin of error. The required sample size was calculated to be 119 [12]. The non-probability, purposive sampling technique was used.

All the information recorded on the questionnaire was analyzed using SPSS version 20.0 (IBM Corp., Armonk, NY, USA). The mean and standard deviation were computed for continuous variables, such as age, weight, height, BMI, and gestational age, whereas frequency and percentages were calculated for categorical variables, such as parity, gravida, mode of delivery, history of macrosomia, previous post-term delivery (>37 weeks), and fetal macrosomia.

Stratification with respect to age, gestational age, gravida, parity, BMI, mode of delivery, and previous history of macrosomia was then evaluated to determine effect modification. The Chi-square test was applied. P-values of ≤0.05 were considered significant.

## Results

In total, 119 participants were included in this study from September 8, 2020, to March 7, 2021. Out of the 119 participants, fetal macrosomia among non-diabetic women was seen in 10 (8.4%) cases. The mean age of patients in our study was 29.80 ± 4.33 years. The mean gestational age was 38.05 ± 1.31 weeks. The mean BMI of participants was 29.17 ± 2.36 kg/m^2^. The detailed demographic characteristics of participants are listed in Table 1. There was no association of fetal macrosomia with age and parity of women. Gestational age, maternal BMI, and previous history of macrosomia had no significant effect on the development of fetal macrosomia.

**Table 1:** Demographic characteristics of study participants. BMI: body mass index; SVD: spontaneous vaginal delivery

Variables		Fetal macrosomia		P-value
		Yes	No	
Age (years)	15–30	05	51	0.846
	31–45	05	58	
Gestational age (weeks)	37–39	06	68	0.882
	>39	04	41	
Gravida	Primigravida	03	17	0.244
	Multigravida	07	92	
Parity	Primiparous	03	28	0.766
	Multiparous	07	81	
BMI (kg/m^2^)	≤25	02	27	0.737
	>25	08	82	
Mode of delivery	SVD	07	35	0.016
	Caesarean	03	74	
History of macrosomia	Yes	00	20	0.138
	No	10	89	

Stratification with respect to age, gestational age, gravidity, parity, BMI, mode of delivery, and previous history of macrosomia was performed to determine significant variables (Tables 2, 3). In our study, SVD was performed in 42 (35.3%) patients, whereas cesarean section was performed in 77 (64.7%) patients. Mode of delivery was the only significant variable with a p-value of 0.016. Seven women with fetal macrosomia (10 cases) had SVD and three underwent cesarean section. A cesarean section was performed due to failed progression of labor (failure of descent). No case of shoulder dystocia occurred in women who underwent vaginal delivery. The fetal outcome was good in terms of APGAR score at one minute and five minutes. APGAR score was 8/10 at one minute in all 10 cases. None of the babies required neonatal intensive care unit admission. Fetal weight ranged from 4 to 4.5 kg in SVDs, while it was >4.5 kg in women undergoing cesarean section due to failure to progress.

**Table 2:** Characteristics of the study participants. BMI: body mass index; SVD: spontaneous vaginal delivery; SD: standard deviation

Variables		No. of patients (n = 119, %)	Mean ± SD
Age (years)	15–30	56 (47.6)	29.80 ± 4.33
	31–45	63 (52.9)	
Gestational period (weeks)	37–39	74 (62.8)	38.05 ± 1.31
	>39	45 (37.8)	
Gravidity	Primigravida	20 (16.8)	
	Multigravida	99 (83.2)	
Parity	Primiparous	31 (26.1)	
	Multiparous	88 (73.9)	
BMI (kg/m^2^)	≤27	29 (24.4)	29.17 ± 2.36
	>27	90 (75.6)	
Mode of delivery	SVD	42 (35.3)	
	Cesarean	77 (64.7)	
Fetal macrosomia among non-diabetic women	Yes	10 (8.4)	
	No	109 (91.6)	

**Table 3:** Chi-square findings for analyzing the hypothesis. BMI: body mass index; SVD: spontaneous vaginal delivery

Variables		Chi-square test
Age (years)	15–30	0.846
	31–45	
Gestational period (weeks)	37–39	0.882
	>39	
Gravida	Primigravida	0.244
	Multigravida	
BMI (kg/m^2^)	≤27	0.737
	>27	
Mode of delivery	SVD	0.016; p-value < 0.05
	Cesarean	
History of macrosomia	Yes	0.138
	No	

## Discussion

In this study, fetal macrosomia was noted in 10 (8.4%) cases among non-diabetic women. The mode of delivery (SVD) was the most common variable in our study with a p-value of <0.05. To our knowledge, this is the first study from Pakistan to determine the number of fetal macrosomia among non-diabetic women. Formerly, a case-control study was conducted at the University of Kinshasa. In this study, 8,268 deliveries were performed between January 2007 to December 2016. Out of the 8,268 deliveries, 308 cases were diagnosed with macrosomia with a frequency of 3.7% [13]. Two case-control studies were performed in Romania. This study was divided into two parts. The first part of the study was performed in 2016 in which they retrieved data from 2,238 pregnant women. Out of 2,238 women, 261 (11.6%) delivered macrosomic babies. In the second part of the study, the authors analyzed data from 2006 in which they extracted data from 2,158 pregnant women. Out of the 2,158 women, 220 (10.1%) women delivered macrosomia babies. Parameters of these two studies were then compared. The study concluded that obesity, weight gain during pregnancy, and previous history of macrosomia increase the risk of delivery of macrosomia baby in the future. In this study, 20 (16.8%) pregnant women had a previous history of macrosomia but none delivered macrosomic babies (p > 0.05), which is contrary to the above-mentioned studies [14].

GDM is a well-known risk factor for fetal macrosomia. The data is scarce with respect to the development of fetal macrosomia among non-diabetic pregnant mothers, especially in developing countries like Pakistan. Neonatal outcomes of macrosomic infants of diabetic and non-diabetic pregnant women were retrospectively reviewed in 2015. The study compared 170 macrosomic fetuses of diabetic mothers with 739 macrosomic fetuses of mothers without diabetes. It concluded that infants of diabetic mothers are mainly delivered by cesarean section, whereas SVD is the common mode of delivery in mothers without diabetes. Out of 170 macrosomic fetuses of diabetic mothers, 35 were delivered by SVD which was then complicated by shoulder dystocia. On the other hand, SVD was the mode of delivery in 70 of non-diabetic mothers out of 739, which was then complicated by shoulder dystocia. The study concluded that both macrosomic babies of diabetic and non-diabetic mothers are at risk of developing morbidity [15]. These findings are comparable with our findings. In our study, SVD was performed in 42 pregnant women. Out of the 42 pregnant women, seven had fetal macrosomia (p < 0.05). This may be the reason that fetal macrosomia remains a challenge for obstetricians in developing countries like Pakistan. The low predictive value of identification of fetal macrosomia is another obstetric challenge for impoverished nations like Pakistan. Pakistan is a resource-limited country where health is not insured, and therefore, pregnant women who are at risk of developing fetal macrosomia do not routinely undergo ultrasound scans due to financial constraints [16]. Second, an ultrasound scan is an operator-dependent investigation; therefore, expertise is needed to establish the diagnosis of fetal macrosomia. Hence, scan results are not standardized and validated in resource-limited countries. Consequently, the prenatal diagnosis of fetal macrosomia is still ambiguous in resource-limited countries [17]. A cross-sectional study was performed to determine the outcomes associated with fetal macrosomia among non-diabetic pregnant women. The study concluded that risk factors associated with fetal macrosomia were advanced maternal age, multiparty, and male gender [18].

The mean birth weight has increased tremendously in various regions of the world over the last several years. This might be due to the delivery of a greater percentage of large for gestational age neonates. Previously, 20 pregnant women without impaired OGTT were investigated at 36 weeks of gestation. Parameters assessed were glucose production, energy expenditure, and insulin resistance. The study concluded that the weight of the fetus mainly relies on maternal glucose production, which, in turn, depends on the extent of insulin resistance. This confirms that insulin resistance plays an important role between maternal weight and fetal macrosomia among non-diabetic women [19]. Obesity is a global problem and maternal obesity directly affects fetal health as well [20]. Earlier, the impact of maternal obesity on fetal growth at different gestational ages was retrospectively reviewed. In total, 356 pregnant non-diabetic women were categorized into the following two groups: pregnant non-diabetic mothers with a BMI of ≥25 kg/m^2^ and pregnant non-diabetic and non-obese mothers. At 19, 30, and 36 gestational weeks, Z-scores of the rate of fetal macrosomia (Abdominal Circumference ≥90th percentile) were assessed between the two groups. The study concluded that Z-scores of the rate of fetal macrosomia were significant in the obese group at 30 and 36 weeks, whereas the difference was not significant between the two groups at 19 weeks of gestation. The study also concluded that maternal obesity prior to pregnancy was linked with fetal macrosomia [21]. We categorized BMI into ≤25 or >25 kg/m^2^. Even though we discovered eight fetal macrosomic fetuses in the obese group, the difference was not statistically significant (p > 0.05), as shown in Table 2.

The prevalence of fetal macrosomia varies worldwide [22]. This might be due to ethnic differences as well as socioeconomic status differences among different populations. In our study, we encountered 10 (8.4%) fetal macrosomia cases among non-diabetic women. A study was conducted in Ethiopia regarding risk factors associated with fetal macrosomia. The prevalence of fetal macrosomia in the study was 19.1% which was more than Iran (2.8%), Nigeria (8%), and Chad (7.6%) [23]. This disparity in incidence shows that ethnicity plays a role in determining fetal macrosomia prevalence [24-26]. The above study was conducted in a private clinic in Mekelle city, Tigray, Ethiopia. Previous studies were conducted in public hospitals in which the prevalence was 6.7%, lower than the study conducted in a private clinic in Ethiopia [27]. This difference in prevalence was likely due to differences in the socioeconomic status of pregnant mothers. This also confirms the possible role of regional variation due to underlying socioeconomic status.

The main drawback of this study was that it was a single-center, cross-sectional study with a small sample size; therefore, we were unable to assess the incidence of fetal macrosomia among non-diabetic women in Pakistan with difficulty in making causal inferences as well. It was a questionnaire-based study that was inexpensive to conduct, especially in a resource-limited country like Pakistan. Further studies with large sample sizes in different healthcare facilities must be conducted to determine the exact prevalence of fetal macrosomia in Pakistan.

## Conclusions

The number of fetal macrosomia cases among non-diabetic women in our study was 10 (8.4%). Because this was a single-center, hospital-based, cross-sectional study, we need to conduct large multicentered randomized controlled studies to identify the actual prevalence of fetal macrosomia in non-diabetic women in our population.

We recommend that awareness programs must be arranged regularly in resource-limited countries to modify dietary and lifestyle attitudes to reduce the occurrence of fetal macrosomia. This will ultimately reduce maternal and fetal complications associated with macrosomia.
